# Lymphatic flow dynamics under exercise load assessed with thoracic duct ultrasonography

**DOI:** 10.1038/s41598-025-99416-8

**Published:** 2025-04-24

**Authors:** Akira Shinaoka, Yoshihiro Kimata

**Affiliations:** 1 Department of Lymphatics and Edematology, Dentistry and Pharmaceutical Science, Okayama University Graduate School of Medicine, 2-5-1 Shikata-cho, Kita-ku, 700-8558 Okayama-shi, Japan; 2 Department of Plastic and Reconstructive surgery, Dentistry and Pharmaceutical Science, Okayama University Graduate School of Medicine, Okayama, Japan; 3 Department of Plastic surgery, Kousei Hospital, Okayama, Japan

**Keywords:** Lymphedema, Lymphatic function, Lymph flow, Chylothorax, Chylous ascites、lymph velocity, Cardiovascular biology, Anatomy, Vascular diseases

## Abstract

The thoracic duct (TD) is the largest lymphatic vessel proximal to the venous system. It undergoes morphological changes in response to lymph flow from the periphery, with automatic contraction controlling the dynamics to propel lymph toward the venous system. Recent advancements in ultrasonography have facilitated non-invasive observations of the TD’s terminal, including its valve and wall motions. Observations of TD movements allow predictions of lymphatic flow dynamics. However, no studies have yet documented the changes in the TD under exercise-induced lymph flow enhancement in humans. Here, using 18-MHz high-frequency ultrasonography, we demonstrate for the first time that the TD diameter significantly expands under exercise load. This study analyzed 20 participants; the maximum TD diameters at rest and post-exercise were 2.69 ± 1.06 mm and 3.41 ± 1.32 mm, respectively (*p* = 0.00000056). While various methods exist for observing the TD, our approach—dynamically monitoring the TD diameter using sonography in real time and correlating it with lymphatic flow dynamics—offers a novel contribution.

**Supplementary Information:**

The online version contains supplementary material available at 10.1038/s41598-025-99416-8.

## Introduction

The thoracic duct (TD) is the largest vessel in the lymphatic system, with a diameter ranging from 2 to 4.8 mm^1^. The lymphatic system begins at the lymphatic capillaries and progresses through pre-collecting lymphatic vessels, collecting vessels, and finally the TD (or right lymphatic duct) before merging with the venous system near the venous angles, which is the junction of the subclavian and internal jugular veins. The TD handles lymph flow from 75% of the body, encompassing the left side, lower body, and gastrointestinal tract, with nearly 97% of these convergences occurring at the left venous angle^2^.

The peripheral lymphatic pumping system comprises several components, with the contractions of the collecting vessels being the most important. These vessels, rich in lymphatic muscle cells, actively promote central lymph flow through repeated automatic contractions^3^. Similarly to the venous system, skeletal muscle pressure in the lower limbs acts as a passive factor in enhancing lymph flow. During lymphography, activities like walking exercises are employed to stimulate lymph flow^4,5^. Recent studies have indicated that respiratory movements^6^and dietary loads can also affect lymph flow^7^.

Although the TD wall passively changes due to these peripheral lymphatic pressures^8^, it has also been demonstrated that the human TD executes automatic contraction movements^9^. It is reported that these automatic lymphatic contractions are influenced by factors such as nitric oxide, which plays a critical role in reducing the spontaneous transient depolarizations of the pacemaker activity in the TD^10–12^. Consequently, the TD wall experiences both passive distension from peripheral lymphatic pressure and active contractions driven by lymphatic muscle, propelling lymph toward the venous system.

There are several methods for observing the TD, including radionuclide imaging, magnetic resonance imaging (MRI), ultrasonography, and fluorescent lymphangiography. Radionuclide imaging allows for TD visualization via tracer absorption from the intestines following oral intake of 123-iodine-labeled beta-methyl branched-chain fatty acids^13,14^. Although selective and convenient, this method is limited by the low resolution of gamma cameras, which hampers detailed anatomical observations. Fluorescent dyes such as indocyanine green, when injected into the interstitial space, are selectively taken up by the lymphatic system and visible at the central TD. However, their penetration is limited to a tissue depth of 1–2 cm, restricting their use to specialized procedures like endoscopy^15^. MRI, using heavily T2-weighted images with long repetition and echo times, offers non-invasive observation of the TD, albeit with lengthy imaging durations^16–19^.

Recent advancements in ultrasonography have made the TD and its terminal parts more accessible for observation. Pathological changes, such as morphological abnormalities of the TD and physiological changes linked to heart disease, dietary intake, and respiration, have been documented^20–23^. However, to our knowledge, there are no reports on real-time changes in the TD induced by exercise load using sonography.

In this study, we aim to elucidate the human TD wall movement response to enhanced lymphatic flow conditions induced by exercise and to gather baseline data on physiological changes in the TD under exercise load conditions using high-frequency ultrasonography.

## Results

### Participants

Table 1 shows participant background data. All data are indicated with mean ± standard deviation. This study included 8 men and 12 women with average age, height, and weight of 39.60 ± 9.37 y, 161.40 ± 7.69 cm, and 51.40 ± 10.00 kg, respectively. Eight participants engaged in over 30 min of exercise almost daily, while the rest exercised less frequently. Twelve participants had eaten meals < 3 h before the test. TD observation was possible in all participants, with two individuals having two TD vein junction sites. We observed only one site in the remaining 18 participants with convergence into the internal jugular vein (IJV) near the venous angle. The terminal ends of the TD were not closed at any time for all participants.

**Table 1:** Background of participants.

Variable	*n* = 20
Men	40
Age, y	39.6 ± 9.37
Height, cm	161.4 ± 7.69
Weight, kg	51.4 ± 10.0
Daily 30-min exercise routine	40
Time after meal < 3 h	60

Data are either mean ± standard deviation or percentages.

## Endpoints

**Fig. 1:**
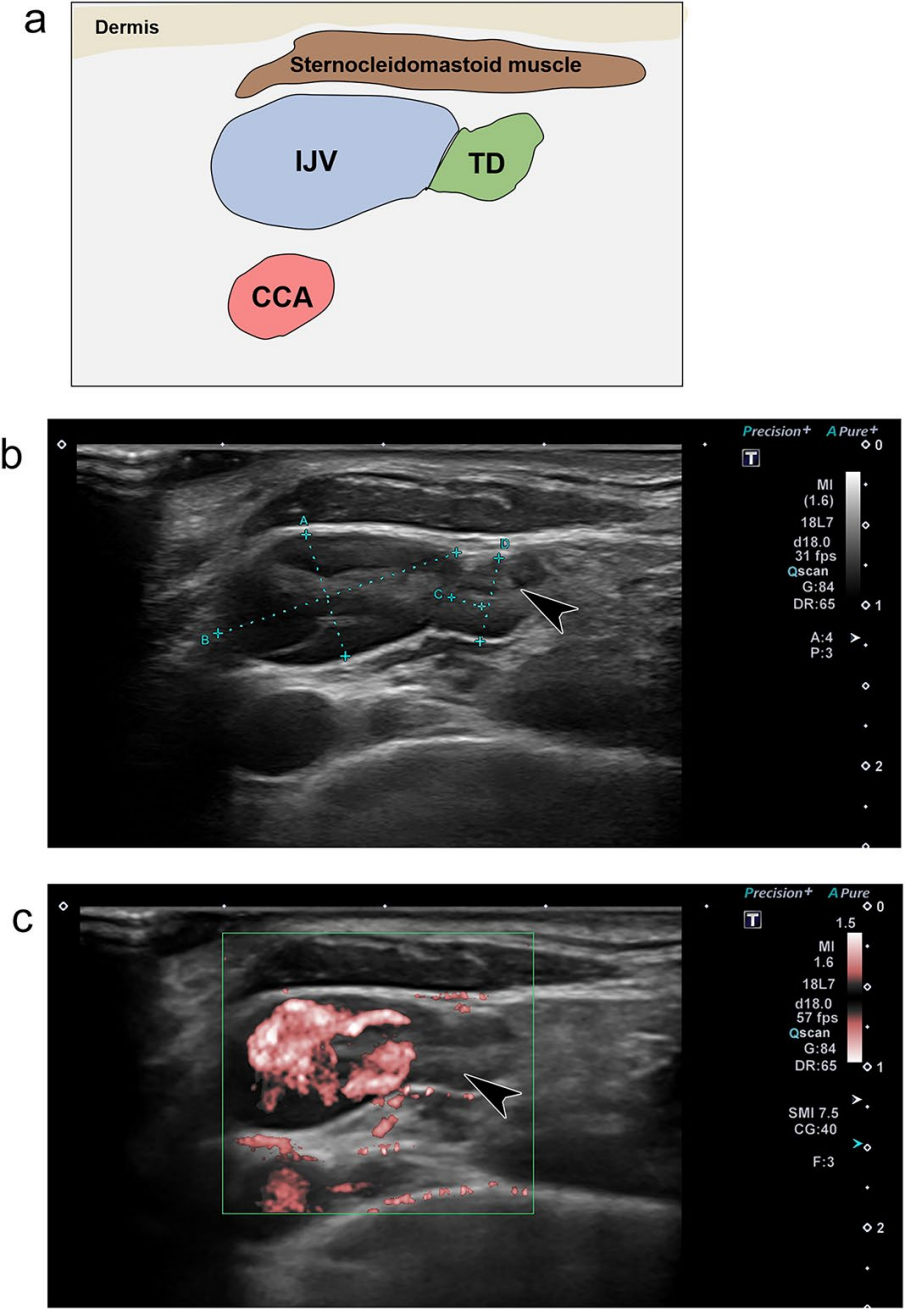
Ultrasonographic images of the thoracic duct (TD). **a** Schematic. **b** The anastomosis site between the TD and the internal jugular vein (IJV) at the left venous angle. **c** Supermicrovascular imaging showing no detectable flow in the TD. The same cross-section is also depicted in Supplementary Video 2. The arrowhead indicates the valve of TD. CCA, common carotid artery.

**Fig. 2:**
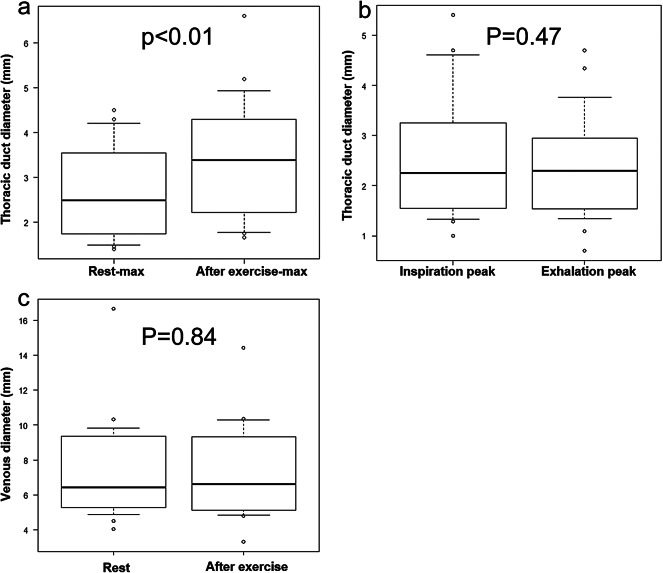
Plots of factors affecting TD diameter. **a** The maximum diameter at rest is 2.7 ± 1.1 mm, which increases after exercise to 3.4 ± 1.3 mm, and the difference is significant (*p* = 0.00000056). **b** The TD diameter is 2.6 ± 1.2 mm and 2.4 ± 1.1 mm at inspiration and exhalation, respectively. No significant differences were observed. **c** The maximum diameter of the IJV at rest is 7.41 ± 2.9 mm, and during exercise, it is 7.47 ± 2.69 mm, with no significant difference observed (*p* = 0.84).

The TD was observed around the left venous angle and underwent slow expansion and contraction in all participants (Fig. 1 and Video 1). The maximum and minimum diameters at rest were 2.69 ± 1.06 mm and 2.25 ± 1.02 mm, respectively. The maximum and minimum diameters after exercise were 3.41 ± 1.32 mm and 2.46 ± 1.24 mm, respectively. The change in diameter post-exercise was 0.72 ± 0.45 mm; the maximum diameters at rest and post-exercise differed significantly (*p* = 0.00000056). During inhalation and exhalation, the diameter was 2.56 ± 1.27 mm and 2.40 ± 1.07 mm, respectively. The maximum diameter of the IJV at rest was 7.41 ± 2.92 mm, and during exercise, 7.47 ± 2.69 mm, with no significant difference observed (*p* = 0.84) (Fig. 2). All boxplots represented the sample minimum, the lower quartile or first quartile, the median, the upper quartile or third quartile, and the sample maximum.

## Factors affecting the increase in TD diameter post-exercise

**Fig. 3:**
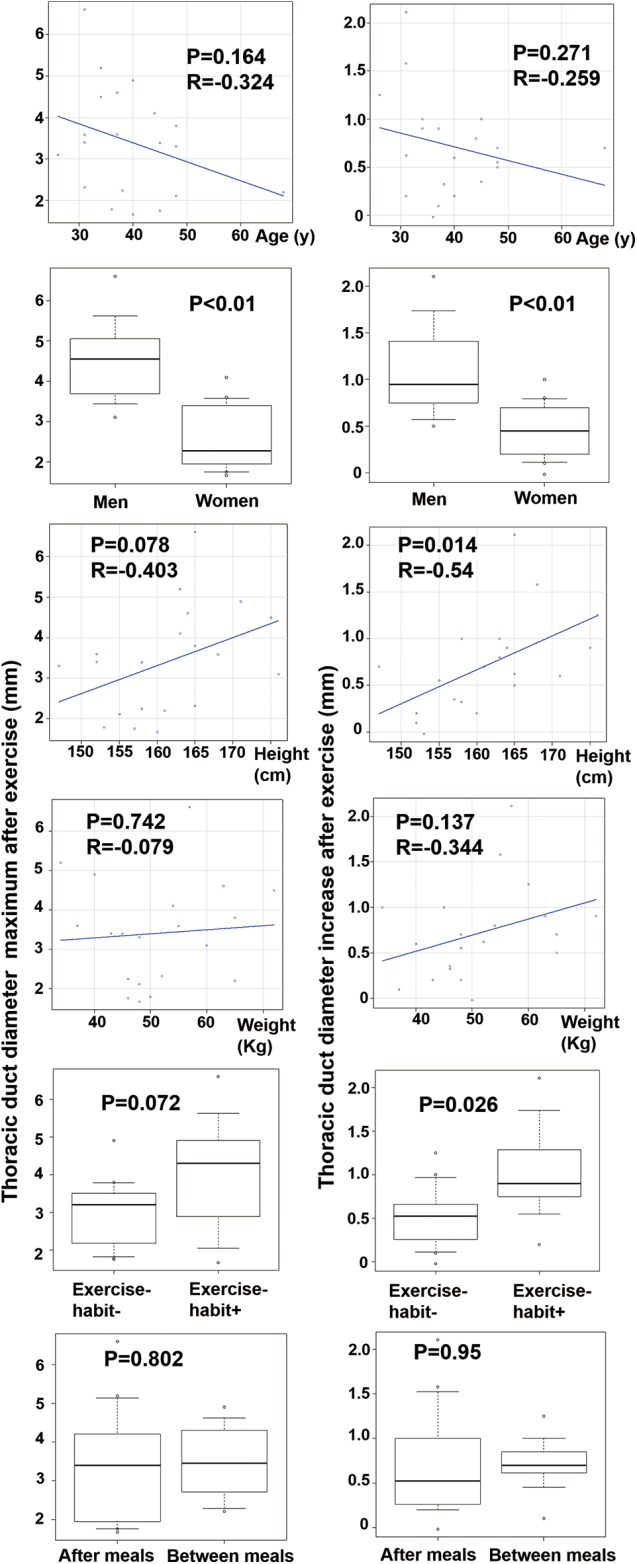
Plots of analytic results for factors influencing TD maximum diameter post-exercise and increase from rest. Gender, height, and exercise habits are statistically significant; however, age, weight, and recent meals are not.

The relation between the maximum diameter post-exercise and the factors that reflect the end points. No correlation was found between age and the maximum diameter post-exercise (correlation coefficient [r]: −0.32; *p* = 0.16). Similarly, no correlation was observed between age and the change in diameter before and after exercise (*r* = −0.26, *p* = 0.27). Gender and maximum diameter post-exercise had a significant relationship, with men having a larger diameter (4.54 ± 1.10 vs. 2.65 ± 0.85 mm, *p* < 0.001). The men also had significantly larger changes in diameter post-exercise (1.11 ± 0.53 vs. 0.46 ± 0.13 mm, *p* = 0.003). No correlation was found between height and maximum diameter post-exercise (*r* = 0.40, *p* = 0.078). However, a correlation was observed between height and the change in diameter post-exercise, indicating a greater increase in taller individuals (*r* = 0.54, *p* = 0.014). No correlation was found between weight and maximum diameter post-exercise (*r* = 0.079, *p* = 0.74) or the change in diameter post-exercise (*r* = 0.34, *p* = 0.14). No significant relationship was found between exercise habits and the maximum diameter post-exercise (exercise habit reported, 4.06 ± 1.59; no exercise habit reported, 3.00 ± 0.95 mm; *p* = 0.072). However, individuals with an exercise habit had a greater increase in diameter post-exercise (exercise habit, 1.02 ± 0.58; no exercise habit, 0.51 ± 0.36 mm; *p* = 0.026). No significant relationship was found between having eaten recently and the maximum diameter post-exercise (post-meal, 3.34 ± 1.55; fasting, 3.50 ± 0.97 mm; *p* = 0.80), nor did we find a significant difference in the increase post-exercise (post-meal, 0.72 ± 0.63; fasting, 0.71 ± 0.32 mm; *p* = 0.95) (Fig. 3).

## Discussion

This study has shown that the terminal portion of the TD can be consistently observed using 18-MHz high-frequency ultrasonography, which revealed an increase in TD diameter under exercise load conditions.

Among the various methods available for observing the TD, ultrasonography stands out as the most convenient and non-invasive option for bedside examination. Its widespread availability in numerous medical facilities enhances its practical applicability. Furthermore, ultrasonography is particularly adept at safely and easily detecting anatomical and physiological changes in the TD, including valve and wall movements—an ability not shared by other imaging technologies^24,25^. Such changes in the TD have been documented in conditions like heart failure^26,27^and cirrhosis^28^. Observing the TD can be crucial in clinical settings if cardiovascular system abnormalities that alter the TD are associated with pathology. Additionally, common lymphatic dysfunction, such as limb lymphedema, often stems from partial regional disorders and anatomical changes in the lymphatic system, especially in peripheral areas like the pelvis or axilla^29–33^. Changes in flow due to peripheral lymphedema may affect the downstream volumetric flow in the TD^34^, suggesting that ultrasonographic evaluation of the TD could be instrumental in diagnosing upstream lymphatic dysfunctions, including limb lymphedema.

The anatomy of the TD is relatively well understood, and the observations from this study align with existing knowledge. We were able to clearly observe up to the venous angle; however, in some instances, deeper observations were not possible. Therefore, we cannot assert that all junction sites of the TD with veins were observed. Nonetheless, with advancements in ultrasonography technology, a more comprehensive evaluation may become feasible. Observed TD sizes varied significantly across individuals, genders, and body sizes, and the extent of exercise-induced changes also varied considerably. This suggests that using TD size as an independent clinical indicator may be challenging. Nevertheless, it is possible to measure changes in duct size due to physiological variations, such as those induced by exercise, and to assess an individual’s lymph dynamics by measuring the baseline duct size prior to exercise. This pilot study, which targeted only healthy individuals, indicates that it may be possible to establish a normal duct size range by observing patients with diseases that may reduce lymph flow.

In this study, exercise was expected to reliably increase lymphatic flow, and indeed, the TD diameter increased due to the exercise load. Although it remains uncertain whether Hagen-Poiseuille’s law, which governs the basic physics of laminar flow in small vessels, applies perfectly to the TD, it has already been demonstrated in rat mesenteric lymphatics that Hagen-Poiseuille’s law was applicable in 85% of contraction cycles in vivo^11^. Thus, in this study of healthy humans, an increase in TD diameter due to increased preload pressure from exercise likely indicates an increase in flow volume within the TD. While this study did not explore the molecular mechanisms behind the increase in TD diameter, it was anticipated that endothelial factors such as nitric oxide responded to the increased lymphatic pressure or shear stress, causing lymphatic muscle distension and an increase in TD diameter.

This study encountered some limitations, including a small sample size comprised solely of healthy individuals, which restricts the broad applicability of the findings. Additionally, the lack of disease-specific models, the presence of uncontrolled influencing factors, the reliance on inferred rather than directly measured venous pressure, and the inherent operator dependency and resolution limits of ultrasonography also pose challenges. Addressing these limitations in future research could remarkably enhance our understanding of TD physiology and refine the diagnostic and therapeutic approaches for lymphatic disorders.

## Conclusion

This study showed that cervical TD observation is feasible in an 18-MHz ultrasonography examination, capturing real-time changes such as valve movements and peristaltic motions. The results suggest the possibility of estimating changes in lymph flow of the TD by measuring the TD diameter.

## Materials and methods

This cohort and pilot observational study included 20 healthy individuals. The Okayama University Ethics Committee approved this study (2211-025), and experiments were conducted in accordance with the guidelines of the Declaration of Helsinki Principles. All participants provided informed consent and signed a written informed consent statement prior to participation in the study. We recorded participant background information, which included gender, age, height, weight, exercise habits, and time since eating. The study spanned December 2022 to March 2023 and recruited volunteers aged between 18 and 80 years.

**Fig. 4:**
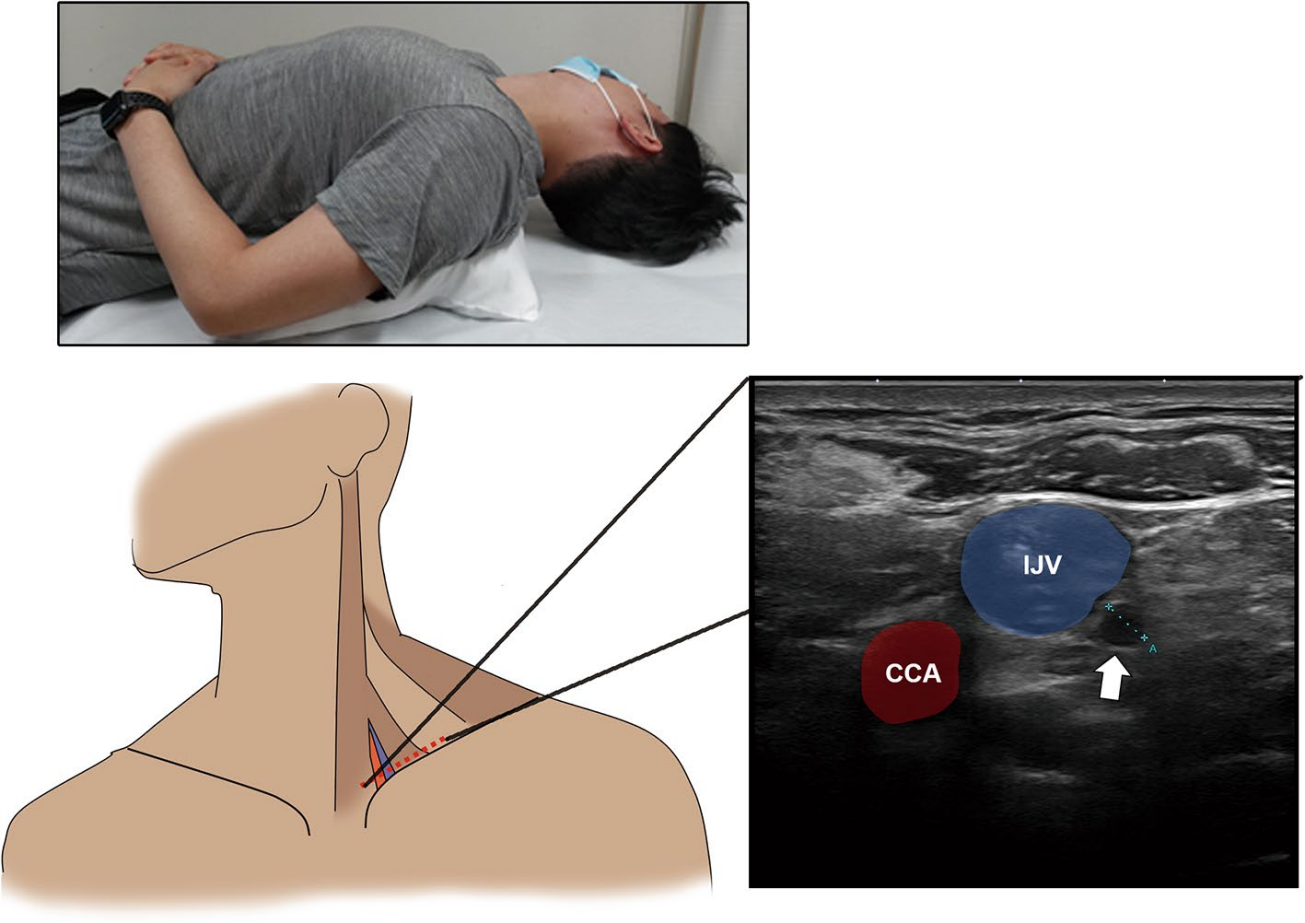
Participant position for the sonography examination. Participants are positioned supine with their chin slightly raised, face tilted approximately 30° to the right side, and with a pillow inserted under the scapula for support. The sonographic probe is positioned perpendicular to the skin and parallel to the clavicle (). The sonographic image shows the short axes of the CCA (), IJV (), and TD ().

The primary endpoints were the details and movements of the TD, observed using a widely available high-frequency probe (18L7 PLU-1204BT; Canon Medical Systems, Ootahara, Japan). We also investigated whether changes in TD diameter could be detected after exercise loading or abdominal breathing. We used the Aplio a/Verifica (Canon Medical Systems, Ootahara, Japan) ultrasonography device with an 18-MHz linear electronic probe. The echo gain was set to the middle of all the observations. The focus point was set to 1 and adjusted to the IJV depth. The dynamic range remained constant throughout the trial. The participants were positioned supine with the chin slightly raised and the face tilted approximately 30° towards the right side for optimal viewing, and a pillow was inserted under the shoulder blades (Fig. 4). The observer sat on the participant’s right side. The probe was positioned perpendicular to the skin and parallel to the clavicle (Fig. 4). The TD was identified by its vascular structure merging into the IJV near the venous angle, the absence of observable flow even with low-speed color Doppler (Superb Microvascular Imaging), and the movement of valve structures (Video 2)^19^. The observation site was standardized to a level where the TD could be observed on a single axis and circularly, with images compared within the same participant to ensure that a similar level was imaged along the anatomical landmarks. In this study, image data to confirm endpoints was only snapshot, not video data. A third party (R.N. and Y.H. with 20 and 10 years of experience in sonography, respectively) ensured imaging consistency.

The study protocol involved obtaining consent, conducting interviews, and commencing observations after sufficient rest. During rest, the participants were instructed to breathe naturally and avoid talking. Images of the maximum and minimum automatic TD movements were captured at rest. Subsequently, the participants were instructed to perform deep abdominal breathing in the rest position, during which TD images were captured at inhalation and exhalation peaks. After imaging at rest, participants performed treadmill walking at 2 km/h for 15 min. Ultrasonography was resumed immediately after the exercise load. Imaging commenced at the same height as during rest, capturing images at the maximum and minimum of the TD’s pumping movements.

We statistically compared continuous variables between the before and after groups using the *t*-test (paired, two-tailed) and calculated Pearson’s product-moment correlation coefficient to examine the relationship between continuous variables. No statistical sample size calculations were conducted. However, from the sample size of 20 participants, we observed a post hoc power of 57% to detect differences in the mean of 3.40 mm and 2.69 mm, respectively, for maximum TD diameter, assuming a common standard deviation of 1.43 mm. Statistical analysis was performed using the R-based software EZR (version 1.41) (https://www.jichi.ac.jp/saitama-sct/SaitamaHP.files/download.html)^35^. The statistical significance level was set at 0.05.

**a** Schematic; **b** the anastomosis site between the TD and the internal jugular vein (IJV) at the left venous angle; **c** supermicrovascular imaging showing no detectable flow in the TD. The same cross-section is also depicted in Supplementary Video 2. The arrowhead indicates the valve of TD. CCA, common carotid artery.

## Electronic supplementary material

Below is the link to the electronic supplementary material.

Supplementary Material 1

Supplementary Material 2

## Data Availability

The data that support the findings of this study are available from the corresponding author, [A.S.], upon reasonable request.
